# Low Free Testosterone and Prostate Cancer Risk: A Collaborative Analysis of 20 Prospective Studies

**DOI:** 10.1016/j.eururo.2018.07.024

**Published:** 2018-11

**Authors:** Eleanor L. Watts, Paul N. Appleby, Aurora Perez-Cornago, H. Bas Bueno-de-Mesquita, June M. Chan, Chu Chen, Barbara A. Cohn, Michael B. Cook, Leon Flicker, Neal D. Freedman, Graham G. Giles, Edward Giovannucci, Randi E. Gislefoss, Graeme J. Hankey, Rudolf Kaaks, Paul Knekt, Laurence N. Kolonel, Tatsuhiko Kubo, Loïc Le Marchand, Robert N. Luben, Tapio Luostarinen, Satu Männistö, E. Jeffrey Metter, Kazuya Mikami, Roger L. Milne, Kotaro Ozasa, Elizabeth A. Platz, J. Ramón Quirós, Harri Rissanen, Norie Sawada, Meir Stampfer, Frank Z. Stanczyk, Pär Stattin, Akiko Tamakoshi, Catherine M. Tangen, Ian M. Thompson, Konstantinos K. Tsilidis, Shoichiro Tsugane, Giske Ursin, Lars Vatten, Noel S. Weiss, Bu B. Yeap, Naomi E. Allen, Timothy J. Key, Ruth C. Travis

**Affiliations:** a Cancer Epidemiology Unit, Nuffield Department of Population Health, University of Oxford, Oxford, UK; b Department for Determinants of Chronic Diseases, National Institute for Public Health and the Environment (RIVM), Bilthoven, The Netherlands; c Department of Gastroenterology and Hepatology, University Medical Centre, Utrecht, The Netherlands; d Department of Epidemiology and Biostatistics, Imperial College London, London, UK; e Department of Social & Preventive Medicine, University of Malaya, Kuala Lumpur, Malaysia; f Department of Epidemiology and Biostatistics, University of California-San Francisco, San Francisco, CA, USA; g Department of Urology, University of California-San Francisco, San Francisco, CA, USA; h Public Health Sciences Division, Program in Epidemiology, Fred Hutchinson Cancer Research Center, Seattle, WA, USA; i Child Health and Development Studies, Public Health Institute, Berkeley, CA, USA; j Division of Cancer Epidemiology and Genetics, U.S. National Cancer Institute, Bethesda, MD, USA; k School of Medicine, University of Western Australia, Perth, Western Australia, Australia; l Western Australian Centre for Health and Ageing, Centre for Medical Research, University of Western Australia, Perth, Western Australia, Australia; m Cancer Epidemiology and Intelligence Division, Cancer Council Victoria, Melbourne, Victoria, Australia; n Centre for Epidemiology and Biostatistics, Melbourne School of Population and Global Health, The University of Melbourne, Victoria, Australia; o Department of Nutrition, Harvard T.H. Chan School of Public Health, Boston, MA, USA; p The Channing Division of Network Medicine, Brigham and Women's Hospital and Harvard Medical School, Boston, MA, USA; q Cancer Registry of Norway, Institute for Epidemiological Cancer Research, Oslo, Norway; r Division of Cancer Epidemiology, German Cancer Research Centre, Heidelberg, Germany; s National Institute for Health and Welfare, Helsinki, Finland; t University of Hawaii Cancer Center, Honolulu, HI, USA; u Department of Preventive Medicine and Community Health, University of Occupational and Environmental Health, Kitakyushu, Japan; v Strangeways Research Laboratory, Department of Public Health and Primary Care, University of Cambridge, Cambridge, UK; w Finnish Cancer Registry, Institute for Statistical and Epidemiological Cancer Research, Helsinki, Finland; x Department of Public Health Solutions, National Institute for Health and Welfare, Helsinki, Finland; y Department of Neurology, University of Tennessee Health Science Center, Memphis, TN, USA; z Department of Urology, Kyoto Prefectural University of Medicine Graduate School of Medical Science, Kyoto, Japan; aa Department of Epidemiology, Radiation Effects Research Foundation, Hiroshima, Japan; bb Department of Epidemiology, Johns Hopkins Bloomberg School of Public Health, Baltimore, MD, USA; cc Public Health Directorate, Asturias, Spain; dd Epidemiology and Prevention Group, Center for Public Health Sciences, National Cancer Center, Tokyo, Japan; ee Division of Reproductive Endocrinology and Infertility, Keck School of Medicine of the University of Southern California, Los Angeles, CA, USA; ff Department of Surgical Sciences, Uppsala University, Uppsala, Sweden; gg Department of Public Health, Hokkaido University Graduate School of Medicine, Sapporo, Japan; hh SWOG Statistical Center, Fred Hutchinson Cancer Research Center, Seattle, WA, USA; ii Cancer Prevention Program, Public Health Sciences Division, Fred Hutchinson Cancer Research Center, Seattle, WA, USA; jj Cancer Therapy and Research Center, University of Texas Health Science Center at San Antonio, TX, USA; kk Department of Hygiene and Epidemiology, University of Ioannina School of Medicine, Ioannina, Greece; ll Department of Nutrition, Institute of Basic Medical Sciences, University of Oslo, Oslo, Norway; mm Department of Preventive Medicine, University of Southern California, Los Angeles, CA, USA; nn Department of Public Health, Faculty of Medicine, Norwegian University of Science and Technology, Trondheim, Norway; oo Department of Epidemiology, School of Public Health, University of Washington, Seattle, WA, USA; pp Department of Endocrinology and Diabetes, Fiona Stanley Hospital, Perth, Western Australia, Australia; qq Clinical Trial Service Unit and Epidemiological Studies Unit, Nuffield Department of Population Health, University of Oxford, Oxford, UK; rr Department of Epidemiology, Harvard T.H. Chan School of Public Health, Boston, MA, USA

**Keywords:** Androgens, Pooled analysis, Prospective studies, Prostate cancer, Sex hormones, Testosterone, Epidemiology

## Abstract

**Background:**

Experimental and clinical evidence implicates testosterone in the aetiology of prostate cancer. Variation across the normal range of circulating free testosterone concentrations may not lead to changes in prostate biology, unless circulating concentrations are low. This may also apply to prostate cancer risk, but this has not been investigated in an epidemiological setting.

**Objective:**

To examine whether men with low concentrations of circulating free testosterone have a reduced risk of prostate cancer.

**Design, setting, and participants:**

Analysis of individual participant data from 20 prospective studies including 6933 prostate cancer cases, diagnosed on average 6.8 yr after blood collection, and 12 088 controls in the Endogenous Hormones, Nutritional Biomarkers and Prostate Cancer Collaborative Group.

**Outcome measurements and statistical analysis:**

Odds ratios (ORs) of incident overall prostate cancer and subtypes by stage and grade, using conditional logistic regression, based on study-specific tenths of calculated free testosterone concentration.

**Results and limitations:**

Men in the lowest tenth of free testosterone concentration had a lower risk of overall prostate cancer (OR = 0.77, 95% confidence interval [CI] 0.69–0.86; *p* < 0.001) compared with men with higher concentrations (2nd–10th tenths of the distribution). Heterogeneity was present by tumour grade (*p*_het_ = 0.01), with a lower risk of low-grade disease (OR = 0.76, 95% CI 0.67–0.88) and a nonsignificantly higher risk of high-grade disease (OR = 1.56, 95% CI 0.95–2.57). There was no evidence of heterogeneity by tumour stage. The observational design is a limitation.

**Conclusions:**

Men with low circulating free testosterone may have a lower risk of overall prostate cancer; this may be due to a direct biological effect, or detection bias. Further research is needed to explore the apparent differential association by tumour grade.

**Patient summary:**

In this study, we looked at circulating testosterone levels and risk of developing prostate cancer, finding that men with low testosterone had a lower risk of prostate cancer.

## Take Home Message

We found that men with low circulating free testosterone had a 23% reduced risk of overall prostate cancer, but there was some evidence that these men had an increased risk of developing high-grade disease.

## 1 Introduction

Experimental and clinical evidence implicates testosterone in the aetiology of prostate cancer. Nearly all metastatic prostate tumours overexpress the androgen receptor, and androgen deprivation therapy is the mainstay treatment approach for many prostate tumours [1]. Two large randomised controlled trials of 5α-reductase inhibitors (which block the conversion of testosterone to the more biologically active dihydrotestosterone [DHT]) showed a reduction in prostate cancer risk [2], [3], [4]. Genome-wide association studies and animal models also support an association between androgens and risk [5], [6], [7], [8].

Despite the strong biological evidence of an association between testosterone concentration and prostate cancer risk, previous epidemiological studies have not found evidence of an association [9]. This may be because the association is nonlinear; variations across the normal range of circulating testosterone may not lead to alterations in prostate growth because the stimulation of prostatic androgen receptors may remain relatively constant, due to relatively constant intraprostatic DHT concentrations and/or saturation of the androgen receptors [10], [11]. However, when the supply of testosterone to the prostate is abnormally low, prostate growth may decrease [12], [13]. Therefore, we hypothesised that men with very low circulating testosterone concentrations may have a reduced risk of prostate cancer but that, above these low concentrations, prostate cancer risk is not associated with further increases in circulating testosterone concentrations. Less than 2% of testosterone circulates unbound to carrier proteins or “free”, and is able to pass out of the blood into the prostate tissue [14]; therefore, the focus of our analysis was on free testosterone.

The Endogenous Hormones, Nutritional Biomarkers and Prostate Cancer Collaborative Group (EHNBPCCG) is a pooled individual participant dataset of prospective studies and prostate cancer risk. A previous analysis by this group found no association between prediagnostic androgen concentrations and prostate cancer [9]. However, this dataset has since been expanded to include almost double the number of prostate cancer cases and now comprises 20 prospective studies with a total of 6933 cases and 12 088 matched controls with calculated free testosterone data. This large dataset now provides sufficient power to examine whether men with very low concentrations of circulating free testosterone have a reduced risk of prostate cancer.

## 2 Patients and methods

### 2.1 Data collection

Individual participant data were available from 20 prospective studies by dataset closure on 31 August 2017. Principal investigators were invited to contribute data to the EHNBPCCG if they had published or unpublished data on concentrations of endogenous hormones and/or nutritional biomarkers from blood samples collected prior to the diagnosis of prostate cancer. Studies were identified using literature search methods from computerised bibliographic systems and by discussion with collaborators, as described previously [9]. Data were harmonised in a central database. Studies were eligible for the current individual participant analysis if they had prospective data on prediagnostic circulating concentrations of testosterone and sex hormone-binding globulin (SHBG), from which an estimate of free testosterone concentration could be calculated. Participating studies are listed in Supplementary Table 1. Further details of data collection and processing are provided in the Supplementary material.

Principal investigators were asked to provide data on prostate cancer case or noncase status, and if applicable, a matched-set identifier. Data were also supplied on participant and tumour characteristics, circulating concentrations of total testosterone, SHBG, as well as other biomarkers that may be potential confounders or sources of bias (prostate-specific antigen [PSA] at blood collection, insulin-like growth factor-I [IGF-I] and C-peptide).

### 2.2 Study design

The majority of the studies were matched case-control studies nested within either prospective cohort studies or randomised trials. Four studies were cohort or case-cohort analyses. To apply a consistent statistical approach across all studies, the cases from the case-cohort studies were matched to up to four participants who were free of prostate cancer at the age at diagnosis of the case on the basis of our minimal matching criteria (Supplementary Table 2).

Each study individually obtained ethical approval; therefore, separate ethical approval for this secondary reanalysis of data was not necessary. Details of participant recruitment, study design, and case ascertainment are summarised in Supplementary Table 1 and assay details in Supplementary Table 3.

### 2.3 Data processing

Free testosterone concentrations were calculated from total testosterone and SHBG concentrations using the law of mass action [15], [16], assuming a constant albumin concentration of 43 g/l. Prostate cancer cases were defined as early stage if they were tumour-node-metastasis (TNM) stage ≤T2 with no reported lymph node involvement or metastases (stage I–II), and advanced stage if they were TNM stage T3 or T4 and/or N1+ and/or M1 (stage III–IV). Aggressive disease was categorised as “no” for TNM stage ≤T3 with no reported lymph node involvement or metastases, and “yes” for TNM stage T4 and/or N1+ and/or M1 and/or stage IV disease or death from prostate cancer. Prostate cancer was defined as low-intermediate grade if the Gleason score was <8 or equivalent and high grade if the Gleason score was ≥8. More detail can be found in the Supplementary material and previous publications [9].

### 2.4 Statistical analysis

Conditional logistic regression was used to calculate the odds of prostate cancer diagnosis by hormone concentration. The analyses were conditioned on the matching variables and adjusted for age at blood collection, body mass index (BMI), height, usual alcohol consumption, smoking status, marital status, and education status as categorical variables, with an additional category for missing data, except for age (continuous). As we were interested a priori in the risk for prostate cancer in men with very low free testosterone concentrations, we categorised free testosterone concentrations into study-specific tenths, with cut points defined by the distribution in control participants, to allow for any systematic differences between the studies in assay methods and blood sample types [17], using the highest tenth as the reference category. To explore the association with greater power, these tenths were also grouped (1, 2–4, 5–7, 8–10), with the 8th–10th tenths combined as the reference category. In all further analyses, the 2nd–10th tenths were combined and used as the reference category. Where more than two categories of exposure were compared, variances were used to calculate floating confidence intervals, which facilitate comparisons between any two exposure groups [18], [19].

PSA, IGF-I, and C-peptide concentrations at blood collection were available for subsets of participants. The main analyses of the relationships between low free testosterone and prostate cancer risk were examined in these subsets before and after further adjustment for these variables (log transformed PSA [continuous] and study-specific fifths of IGF-I, and C-peptide concentrations).

Heterogeneity among studies was assessed by comparing the χ^2^ values for models with and without a study × analyte interaction term. Tests for heterogeneity for case-defined factors, in which controls in each matched set were assigned to the category of their matched cases, were obtained by fitting separate models for each subgroup and assuming independence of the odds ratios (ORs) using a χ^2^ test, which is analogous to a meta-analysis. Tests for heterogeneity for non–case-defined factors were assessed with χ^2^ tests of interaction between subgroups and the binary variable.

All tests of statistical significance were two sided, and statistical significance was set at the 5% level. All statistical tests were carried out with Stata statistical software, release 14.1 (StataCorp, College Station, TX, USA). Further details of the statistical analysis can be found in the Supplementary material.

## 3 Results

A total of 20 studies, comprising 6933 cases and 12 088 controls, were eligible for this analysis. Mean age at blood collection in each study ranged from 33.8 to 76.2 yr (overall mean = 59.8 yr, standard deviation [SD] = 11.5 yr), and the year of blood collection ranged from 1959 to 2004. Study participants were predominantly of white ethnic origin (82%). The average time from blood collection to diagnosis was 6.8 yr, the average age at diagnosis was 67.9 yr (SD = 7.2), and most cases were diagnosed between 1995 and 1999 (39%). Prostate cancers were mostly localised (55%) and/or low grade (68%; Table 1). The free testosterone concentration cut points used for each study are shown in Supplementary Table 4. Men in the lowest study-specific tenth of free testosterone were older, and had a higher mean BMI and lower PSA at blood collection than men with higher free testosterone concentrations (Table 2).

**Table 1:** Characteristics of patients with prostate cancer by study a

Study	*N*	Years from blood collection to diagnosis (%)		Age at diagnosis (%)		Year of diagnosis (%)		Stage of disease b (%)			Aggressive disease c (%)			Grade of disease d (%)		
		<5	5+	<65	65+	<1995	1995+	Localised e	Advanced e	*N*/*k*	No e	Yes e	*N*/*k*	Low e	High e	*N*/*k*
ATBC	116	72	28	48	52	100	0	61	39	0	72	28	0	80	20	72
BLSA	112	15	85	19	81	68	32	79	21	53	64	36	38	87	14	15
CARET	298	83	17	38	62	45	55	69	31	28	91	9	28	89	11	83
CHDS	322	0	100	46	54	44	56	80	20	11	85	15	11	99	1	0
EPIC	490	78	22	54	47	1	99	70	30	33	67	33	26	89	12	78
EPIC-Norfolk	76	13	87	8	92	0	100	76	24	8	59	41	4	71	29	13
FMC	166	13	87	22	78	100	0	NA	NA	100	NA	NA	100	NA	NA	13
HHS NBSBWG	84	7	93	67	33	76	24	62	38	21	62	38	21	NA	NA	7
HIMS	319	66	34	0	100	0	100	NA	NA	100	0	100	89	NA	NA	66
HPFS	682	82	18	27	73	11	89	83	17	43	96	4	43	90	10	82
JACC	40	45	55	8	93	55	45	NA	NA	100	NA	NA	100	NA	NA	45
JPHC	201	19	81	18	82	4	97	72	29	25	70	30	23	76	24	19
Janus NBSBWG	491	3	97	59	41	81	19	NA	NA	100	NA	NA	100	NA	NA	3
MCCS	548	41	59	30	70	15	85	91	9	2	98	2	2	86	14	41
MEC	463	94	7	20	80	0	100	NA	NA	100	0	100	94	100	0	94
MMAS	163	20	80	31	69	29	71	NA	NA	100	0	100	98	100	0	20
NSHDC	384	37	63	55	45	5	95	80	20	1	88	12	1	90	10	37
PCPT	1032	23	77	23	77	0	100	98	2	3	99	1	3	95	5	23
PHS	219	30	70	32	68	100	0	78	22	3	70	31	3	91	9	30
PLCO	727	89	11	25	75	0	100	89	11	0	95	5	0	95	5	89

ATBC = Alpha-Tocopherol, Beta-Carotene Cancer Prevention Study; BLSA = Baltimore Longitudinal Study of Aging; CARET = Carotene and Retinol Efficacy Trial; CHDS = Child Health and Development Studies; EPIC = European Prospective Investigation into Cancer and Nutrition; FMC = Finnish Mobile Clinic Health Examination Survey*;* HHS = Helsinki Heart Study; HIMS = Health In Men Study; HPFS *=* Health Professionals Follow-up Study; JACC = Japan Collaborative Cohort Study; JPHC = Japan Public Health Center-based Prospective Study; MCCS = Melbourne Collaborative Cohort Study; MEC = Multiethnic Cohort Study of Diet and Cancer; MMAS = Massachusetts Male Aging Study; NA = not available; NBSBWG = Nordic Biological Specimen Biobank Working Group; NSHDC = Northern Sweden Health and Disease Cohort; PCPT = Prostate Cancer Prevention Trial; PHS = Physicians’ Health Study; PLCO = Prostate, Lung, Colorectal and Ovarian Cancer Screening Trial; TNM = tumour-node-metastasis.

a Percentage value is for cases with an available free testosterone measurement.

b Stage of disease was defined as follows: localised if TNM was T2 or lower with no reported lymph node involvement or metastases, stage II or lower, or equivalent (ie, a tumour that does not extend beyond the prostate capsule); advanced if TNM stage was T3 or T4 and/or N1+ and/or M1, stage III or IV, equivalent (ie, a tumour extending beyond the prostate capsule and/or lymph node involvement and/or distant metastases), or unknown.

c Aggressive disease was defined as T4 and/or N1+ and/or M1, or stage IV disease and/or death from prostate cancer. Overall, 4661 (67%) of case patients had data on disease aggressiveness.

d Histological grade was categorised as low-intermediate grade (Gleason sum <8 or cases coded as well, moderately, or poorly differentiated), high grade (Gleason sum 8+ or cases coded as undifferentiated), or unknown.

e Percentage value is for those with known disease characteristics.

**Table 2:** Characteristics of prostate cancer cases and controls by study-specific tenths of calculated free testosterone concentration

	Cases		Controls	
	1st tenth	2nd–10th tenth	1st tenth	2nd–10th tenth
Age (yr), mean (SD)	62.8 (10.2)	60.5 (10.6)	60.8 (12.3)	59.2 (12.0)
Height (cm), mean (SD)	175.9 (7.6)	175.1 (7.7)	174.6 (8.2)	174.5 (7.7)
BMI (kg/m^2^), mean (SD)	27.5 (4.6)	26.4 (3.6)	27.1 (4.8)	26.3 (3.7)
PSA (ng/ml), mean (SD)	6.63 (32.8)	7.59 (137.0)	1.30 (1.45)	1.54 (2.7)
Smoking status (%)				
Never	30	33	27	27
Ex	48	42	39	38
Current	16	19	24	23
Unknown	6	7	10	11
Alcohol consumption (g/d)				
Nondrinkers	20	18	18	16
<10	27	26	20	21
≥10	32	33	29	30
Unknown	21	23	33	33
Ethnicity (%)				
White	84	84	81	81
African American	7	6	7	6
East Asian	3	5	5	6
Other	2	2	2	2
Unknown	4	3	5	5
Currently married/cohabiting (%)				
Yes	76	74	66	66
No	11	11	10	10
Unknown	14	16	24	24

BMI = body mass index; PSA = prostate-specific antigen; SD = standard deviation.

### 3.1 Associations between calculated circulating free testosterone concentration and prostate cancer risk

**Fig. 1:**
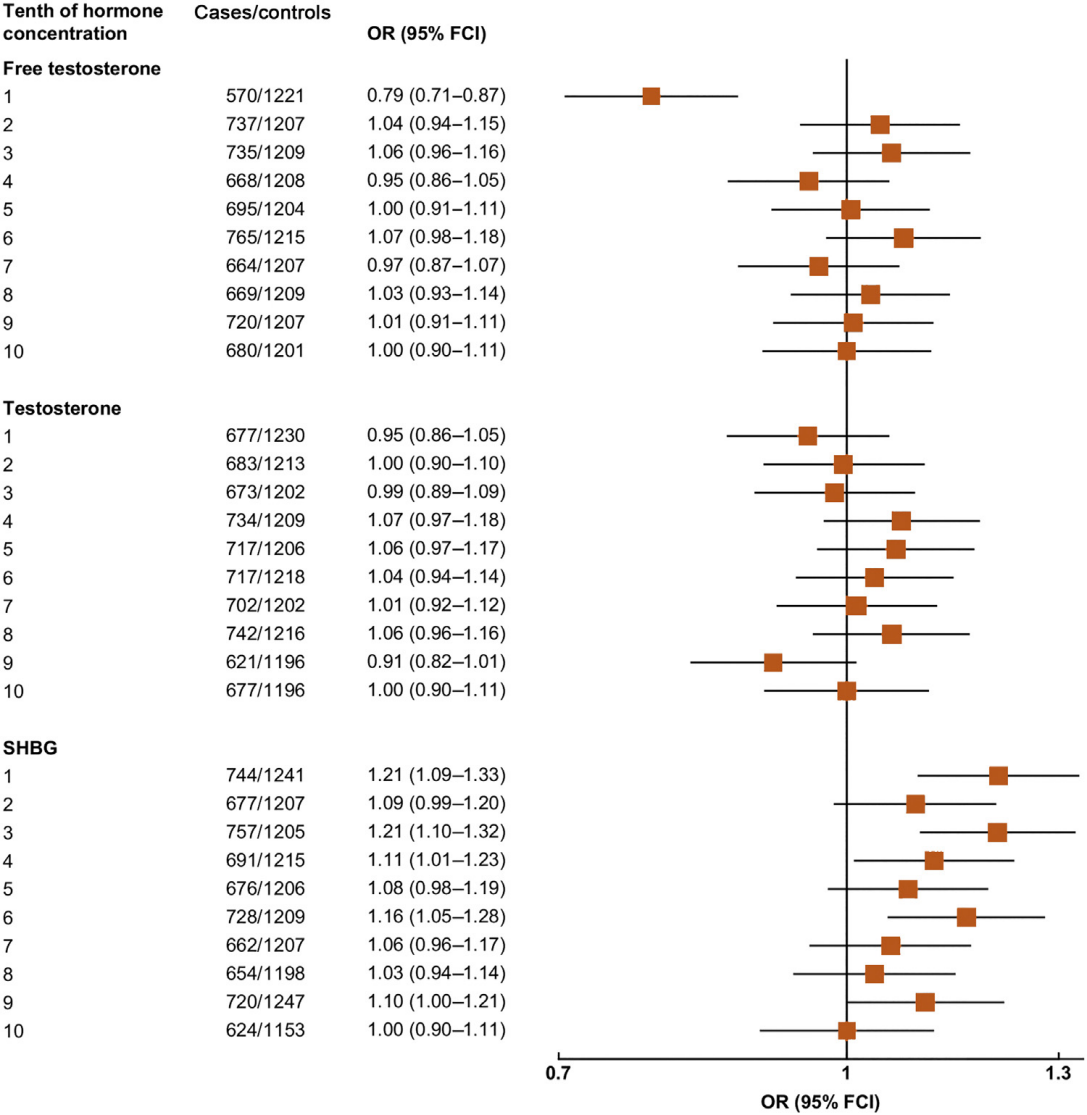
Associations between risk of overall prostate cancer and study-specific tenths of hormone concentrations. Estimates are from logistic regression conditioned on the matching variables and adjusted for age, BMI, height, alcohol intake, smoking status, marital status, and education status. The position of each square indicates the magnitude of the relative risk, and the area of the square is proportional to the amount of statistical information available (inverse of the variance of the logarithm of the relative risk). The length of the horizontal line through the square indicates the 95% floated confidence interval. BMI = body mass index; FCI = floated confidence interval; OR = odds ratio; SHBG = sex hormone–binding globulin.

**Fig. 2:**
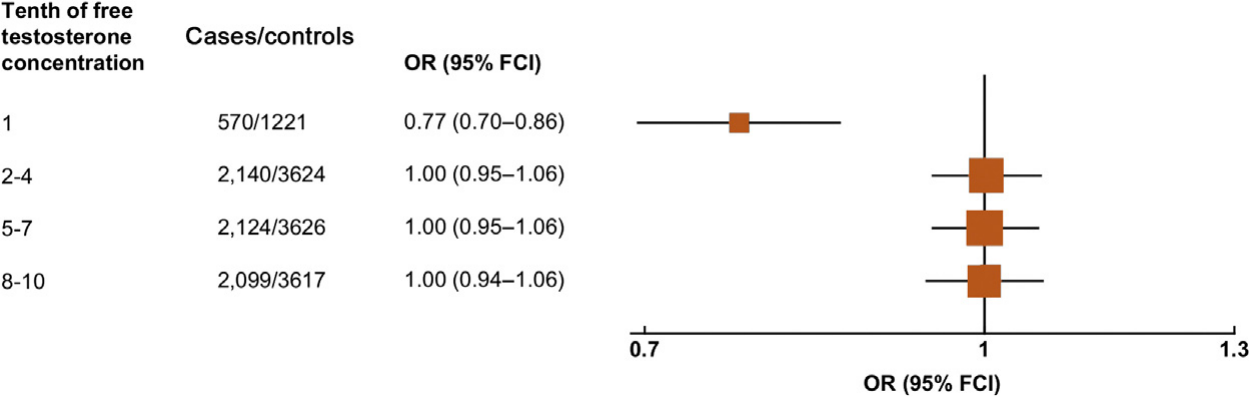
Odds ratio (95% FCIs) for overall prostate cancer associated with study-specific tenths of concentrations of free testosterone. Estimates are from logistic regression conditioned on the matching variables and adjusted for age, BMI, height, alcohol intake, smoking status, marital status, and education status. BMI = body mass index; FCI = floated confidence interval; OR = odds ratio.

**Fig. 3:**
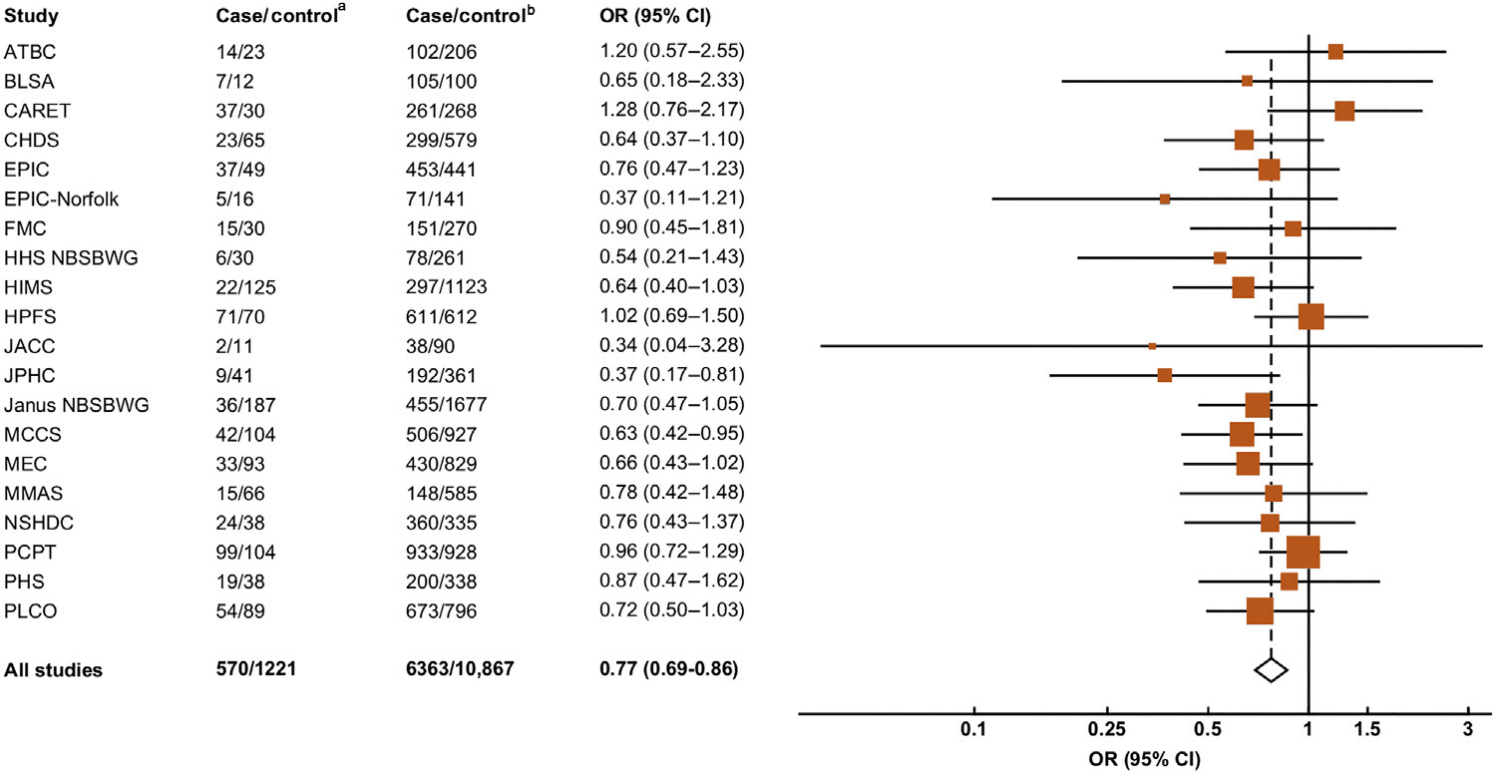
Odds ratio (95% CIs) for overall prostate cancer for the 1st tenth of free testosterone concentration in comparison to the 2nd–10th tenths by study. Estimates are from logistic regression conditioned on the matching variables and adjusted for age, BMI, height, alcohol intake, smoking status, marital status, and education status. ATBC = Alpha-Tocopherol, Beta-Carotene Cancer Prevention Study; BMI = body mass index; BLSA = Baltimore Longitudinal Study of Aging; CARET = Carotene and Retinol Efficacy Trial; CHDS = Child Health and Development Studies; CI = confidence interval; EPIC = European Prospective Investigation into Cancer and Nutrition; FMC = Finnish Mobile Clinic Health Examination Survey*;* HHS = Helsinki Heart Study; HIMS = Health In Men Study; HPFS *=* Health Professionals Follow-up Study; JACC = Japan Collaborative Cohort Study; JPHC = Japan Public Health Center-based Prospective Study; MCCS = Melbourne Collaborative Cohort Study; MEC = Multiethnic Cohort Study of Diet and Cancer; MMAS = Massachusetts Male Aging Study; NBSBWG = Nordic Biological Specimen Biobank Working Group; NSHDC = Northern Sweden Health and Disease Cohort; OR = odds ratio; PCPT = Prostate Cancer Prevention Trial; PHS *=* Physicians’ Health Study; PLCO = Prostate, Lung, Colorectal and Ovarian Cancer Screening Trial. Test of significance (studies without organised screening): *p* < 0.001. Test of heterogeneity between studies without organised screening = χ^2^_17_ = 15.88; *p* = 0.53. Test of significance (studies with organised screening): *p* = 0.16. Test of heterogeneity between studies with organised screening = χ^2^_1_ = 1.29; *p* = 0.26. Test of heterogeneity between studies with and without organised screening = χ^2^_1_ = 0.73; *p* = 0.39. Test of significance (overall): *p* < 0.001. Test of heterogeneity overall = *χ*^2^_19_ = 18.0; *p* = 0.53. ^a^ 1st study-specific tenth of free testosterone. ^b^ 2nd–10th study-specific tenths of free testosterone.

Fig. 1 shows the associations of free testosterone, total testosterone, and SHBG concentrations with overall prostate cancer risk. Men in the lowest tenth of free testosterone had a lower risk of prostate cancer compared with men in any other tenth of the distribution (Fig. 1). We next combined the tenths into a smaller number of categories (1, 2–4, 5–7, 8–10); here, men in the lowest tenth had a 23% lower risk compared with men in the 8th–10th tenth group (Fig. 2). When categories 2nd–10th were combined, the risk estimate remained very similar (OR for 1st vs 2nd–10th category = 0.77, 95% CI 0.69–0.86; *p* < 0.001), with no evidence of heterogeneity between studies (χ^2^_19_ = 18.0; *p* = 0.53; Fig. 3). Two studies (Prostate Cancer Prevention Trial [PCPT] and Prostate, Lung, Colorectal, and Ovarian Cancer Screening Trial) included organised prostate cancer screening (25% of case participants), but there was no evidence of heterogeneity between studies that included organised screening and those that did not (χ^2^_1_ = 0.73; *p* = 0.39).

PSA concentration at blood collection was available for 48% of matched sets. In this subset, men with low free testosterone had a reduced risk of prostate cancer (OR = 0.74; 95% CI = 0.64–0.85); further adjustment for PSA attenuated the association to the null (OR = 0.92; 95% CI 0.76–1.12). In men with data for IGF-I and C-peptide, further adjustment for these analytes made no appreciable difference in the associations (results not shown).

**Fig. 4:**
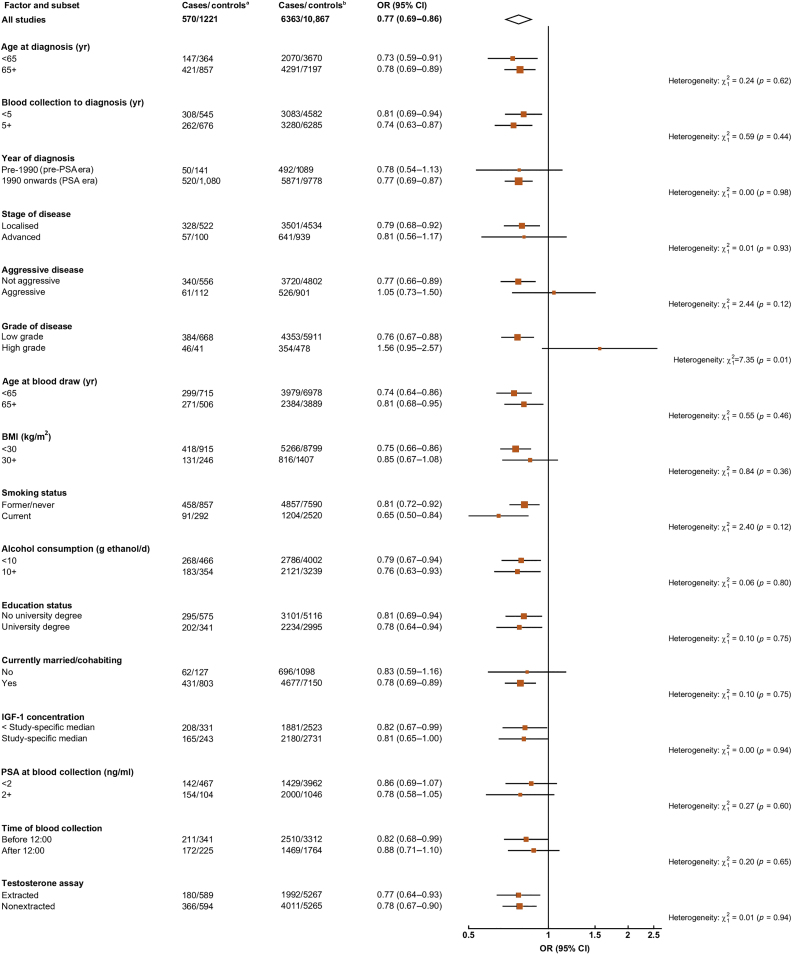
ORs (95% CIs) for prostate cancer associated with free testosterone in the study-specific 1st tenth compared with the 2nd–10th tenths, according to characteristics of cases and controls. Estimates are from logistic regression conditioned on the matching variables and adjusted for age, BMI, height, alcohol intake, smoking status, marital status, and education status. BMI = body mass index; CI = confidence interval; IGF = insulin-like growth factor; OR = odds ratio; PSA = prostate-specific antigen. Tests for heterogeneity for case-defined factors were obtained by fitting separate models for each subgroup and assuming independence of the ORs using a method analogous to a meta-analysis. Tests for heterogeneity for non–case-defined factors were assessed with a χ^2^ test of interaction between subgroup and the binary variable. ^a^ 1st study-specific tenth of free testosterone. ^b^ 2nd–10th study-specific tenths of free testosterone.

There was evidence of heterogeneity by tumour grade (χ^2^_1_ = 7.35; *p*_het_ = 0.01); a low concentration of circulating free testosterone was associated with a reduced risk of low-grade prostate cancer (OR = 0.76; 95% CI 0.67–0.88), while there was a nonsignificantly increased risk of high-grade prostate cancer (OR = 1.56; 95% CI 0.95–2.57; Fig. 4. There was no evidence of heterogeneity in the association by tumour stage, aggressiveness, PSA era, or other characteristics (Fig. 4).

## 4 Discussion

Our results indicate that men in the lowest study-specific tenth of calculated free testosterone concentration have a 23% reduced risk of prostate cancer compared with men with higher concentrations. Above this very low concentration, prostate cancer risk did not change with increasing free testosterone concentration. We also found evidence that this association varied by tumour grade. This is the largest collection of data on hormones and prostate cancer risk available, and is the first large-scale prospective evidence supporting an association between low free testosterone concentrations and prostate cancer risk.

The observed association between low free testosterone and lower prostate cancer risk may be due to a direct biological effect. Across the normal range of circulating free testosterone concentrations, stimulation of prostatic androgen receptors may remain relatively constant, due to stable intraprostatic DHT concentrations and/or saturation of androgen receptors [10], [11]. Therefore, variation across the normal range of circulating free testosterone concentrations may not be associated with a prostate cancer risk. However, when circulating concentrations are very low, reduced androgen receptor signalling may lead to a reduction in prostate cancer risk [10], [11], [20].

An alternative explanation for the main findings may be detection bias. Controls with low free testosterone concentrations had low PSA concentrations at blood collection, and adjustment for PSA concentration in a subset of our dataset attenuated the association of low free testosterone and prostate cancer risk towards the null. However, there was no evidence of heterogeneity in the associations between men diagnosed before and after 1990, before which there was relatively little PSA testing [21]. PSA is partly regulated by the androgen receptor [12], [22]; therefore, it is difficult to disentangle the relationship between these variables in this observational analysis [12], [21], [22].

While there was no evidence of heterogeneity in the association of free testosterone with prostate cancer risk by tumour stage or aggressiveness, there was evidence of heterogeneity in this association by tumour histological grade; a low free testosterone concentration was associated with a lower risk of low-intermediate–grade prostate cancer, and there was a nonsignificantly increased risk of high-grade disease. Although it is possible that this heterogeneity is a chance finding due to the multiple tests conducted and the relatively small number of high-grade tumours, this pattern has been reported previously in the Health Professionals Follow-up Study [23], several clinical case studies [24], [25], [26], and the PCPT and Reduction by Dutasteride of Prostate Cancer Events (REDUCE) trials. These two trials investigated the effect of 5α-reductase inhibitors, which can reduce intraprostatic DHT concentration by approximately 80–90%, on prostate cancer risk [27]. Both trials reported a 23–25% reduction in overall prostate cancer [3], [4]. However, the PCPT reported a 27% increase in high-grade (Gleason grade ≥7) tumours (*n* = 517) [4], and the REDUCE trial reported a 58% increased risk of high-grade (Gleason grade ≥8) tumours (*n* = 48) [3].

There are several possible explanations for the observed heterogeneity in the associations by tumour grade. Prostate tumour grade stays stable over several years [28], suggesting that high-grade tumours develop de novo rather than from the dedifferentiation of low-intermediate–grade tumours. Mechanistically, prostatic androgen-androgen receptor binding is an important modulator of cell differentiation [29]; thus, prostate cells with reduced androgen exposure may be less differentiated and more likely to develop into high-grade tumours [30]. Alternatively, this may be a differential growth response of early low-grade cancer lesions to a low androgen environment. Another possibility is differential detection bias as discussed in relation to PCPT and REDUCE [31], [32], [33], [34], [35], [36]. Owing to the clinical importance of high-grade tumours, this observed heterogeneity by grade, with a possible higher risk of high-grade tumours, requires further investigation.

Our study has a number of limitations. Free testosterone was calculated using the law of mass action [15], [16], which is based on testosterone and SHBG concentrations and assumes a constant albumin concentration. Although this is a commonly used method of estimating free testosterone concentration, it has not been validated within each individual study via equilibrium dialysis [14]. The assay methods used to measure analytes varied, with the majority of studies using nonextraction assays to measure testosterone. While this may introduce some misclassification, this would be expected to be nondifferential and therefore tend to bias any association towards the null. Mass spectrometry is often considered the gold standard method to measure sex hormone concentrations [37], but high-quality immunoassays are able to measure reliably low adult male testosterone concentrations [38], [39]. Although these assays may not be suitable for determining absolute clinical cut points, they are considered appropriate for the determination of relative concentrations within studies [39]. Our study relied on single measurements of testosterone and SHBG, with an average time from blood collection to diagnosis of 6.8 yr, to represent participants’ hormone concentrations over medium to long term. While several studies show that a single measure of these analytes has moderately good reproducibility over periods of up to 1 yr[40], it is unknown whether these measures are reliable over the longer term.

## 5 Conclusions

In summary, the findings from this pooled prospective analysis of 6933 prostate cancer cases and 12 088 controls support the hypothesis that very low concentrations of circulating free testosterone are associated with a reduced risk of prostate cancer. Further research is needed to elucidate whether the association is causal or due to detection bias, and explore the apparent differential association by tumour grade.

  ***Author contributions***: Eleanor L. Watts had full access to all the data in the study and takes responsibility for the integrity of the data and the accuracy of the data analysis.  

*Study concept and design*: Watts, Appleby, Perez-Cornago, Allen, Key, Travis.

*Acquisition of data*: Bueno-de-Mesquita, Chan, Chen, Cohn, Cook, Flicker, Freedman, Giles, Giovannucci, Gislefoss, Hankey, Kaaks, Knekt, Kolonel, Kubo, Le Marchand, Luben, Luostarinen, Männistö, Metter, Mikami, Milne, Ozasa, Platz, Quirós, Rissanen, Sawada, Stampfer, Stanczyk, Stattin, Tamakoshi, Tangen, Thompson, Tsilidis, Tsugane, Ursin, Vatten, Weiss, Yeap, Allen, Key.

*Analysis and interpretation of data*: Watts, Appleby, Perez-Cornago, Allen, Key, Travis.

*Drafting of the manuscript*: Watts.

*Critical revision of the manuscript for important intellectual content*: Watts, Appleby, Perez-Cornago, Bueno-de-Mesquita, Chan, Chen, Cohn, Cook, Flicker, Freedman, Giles, Giovannucci, Gislefoss, Hankey, Kaaks, Knekt, Kolonel, Kubo, Le Marchand, Luben, Luostarinen, Männistö, Metter, Mikami, Milne, Ozasa, Platz, Quirós, Rissanen, Sawada, Stampfer, Stanczyk, Stattin, Tamakoshi, Tangen, Thompson, Tsilidis, Tsugane, Ursin, Vatten, Weiss, Yeap, Allen, Key, Travis.

*Statistical analysis*: Watts, Appleby.

*Obtaining funding*: Key, Travis.

*Administrative, technical, or material support*: None.

*Supervision*: Perez-Cornago, Key, Travis.

*Other*: None.  

***Financial disclosures:*** Eleanor L. Watts certifies that all conflicts of interest, including specific financial interests and relationships and affiliations relevant to the subject matter or materials discussed in the manuscript (eg, employment/affiliation, grants or funding, consultancies, honoraria, stock ownership or options, expert testimony, royalties, or patents filed, received, or pending), are the following: None.  

***Funding/Support and role of the sponsor*****:** Centralised pooling, checking, and data analysis were supported by Cancer Research UK grants C8221/A19170 and C8221/A20986.  

*Acknowledgements:* The authors thank the men who participated in the collaborating studies, the research staff, collaborating laboratories, and funding agencies in each of the studies. Details of funding for the original studies are found in the relevant publications. These include the following: the Intramural Research Program of the National Institute on Aging, National Institute of Health; Eunice Kennedy Shriver National Institute of Child Health and Development; National Institutes of Health and Department of Health and Human Services (grant number: HHSN275201100020C); California Department of Public Health as part of the state-wide cancer reporting program mandated by California Health and Safety Code Section 103885; National Cancer Institute's Surveillance, Epidemiology and End Results Program awarded to the Cancer Prevention Institute of California (grant number: HHSN261201000140C); National Cancer Institute's Surveillance, Epidemiology and End Results Program awarded to the University of Southern California (grant number: HHSN261201000035C); National Cancer Institute's Surveillance, Epidemiology and End Results Program awarded to the Public Health Institute; Centers for Disease Control and Prevention's National Program of Cancer Registries (grant number: HHSN261201000034C); California Department of Public Health (grant number: U58DP003862-01); Cancer Research Fund, under Interagency Agreement #97-12013 (University of California contract #98-00924 V) with the Department of Health Services, Cancer Research Program (grant number: UM1 CA182883); National Institutes of Health/National Cancer Institute (grant numbers: CA167552, CA055075, CA133891, CA141298, CA09001, CA131945, CA34944, CA37429, CA40360, CA097193); National Institutes of Health/National Heart, Lung and Blood Institute (grant numbers: HL26490, HL34595); Dana-Farber Cancer Institute Mazzone Awards Program; National Cancer Institute (grant number U01 CA164973); National Health and Medical Research Council of Australia. EPIC Spain wishes to acknowledge the Regional Governments of Asturias, Andalucia, Navarra, Murcia, and Basque Country for funding. The authors wish to thank the Massachusetts Male Aging Study for contributing data for their analysis.
