# Supplementary material for: Low Free Testosterone and Prostate Cancer Risk: A Collaborative Analysis of 20 Prospective Studies
Source: Eur Urol. 2018 Nov;74(5):585–94. doi: 10.1016/j.eururo.2018.07.024 (PMC6195673; doi:10.1016/j.eururo.2018.07.024)
Supplement: Supplementary file 1 [file mmc1.docx]

**Supplementary material**

**Table of Contents**

**Supplementary methods**

Data collection Page 2-3

Data processing Page 4

Statistical analyses Page 4-6

**Supplementary tables and figures**

Supplementary Tables 1-4 Page 7-10

**References for the supplementary material** Page 11-13

**Supplementary methods**

**Data collection**

Principal investigators were invited to join this collaborative group if they had published or unpublished studies on prostate cancer risk and endogenous sex hormone concentrations that had been determined from blood samples collected before diagnosis. Studies were identified by literature searches of computerised bibliographic systems, including PubMed, Web of Science, Cochrane Library, and CancerLit, and through discussions with colleagues.

Individual participant data were available from 20 prospective studies by dataset closure on August 31^st^, 2017. We included all studies with total testosterone and sex hormone-binding globulin (SHBG) measurements, from which an estimate of free testosterone concentration could be calculated. In total 6,933 cases and 12,088 controls were analysed from the following studies: Alpha-Tocopherol, Beta-Carotene Cancer Prevention Study (ATBC)[1], Baltimore Longitudinal Study of Aging (BLSA)[2, 3], Carotene and Retinol Efficacy Trial (CARET)[4], Child Health and Development Studies (CHDS)[5], European Prospective Investigation into Cancer and Nutrition (EPIC)[6], EPIC Norfolk[7], Finnish Mobile Clinic (FMC)[8], Helsinki Heart Study (HHS)[9, 10], Health Professionals Follow-up Study (HPFS)[11], Health In Men Study (HIMS)[12, 13], Japan Collaborative Cohort Study (JACC)[14], Japan Public Health Center-based prospective study (JPHC)[15], Janus serum bank[9, 16], Melbourne Collaborative Cohort Study (MCCS)[17], Multiethnic Cohort (MEC)[18], Massachusetts Male Aging Study (MMAS)[19], Northern Sweden Health and Disease Cohort (NSHDC)[9], Prostate Cancer Prevention Trial (PCPT)[20], Physicians’ Health Study (PHS)[21], and the Prostate, Lung, Colorectal and Ovarian Cancer Screening Trial (PLCO)[22, 23].

The characteristics of these studies in the collaborative analyses are found their original publications and are summarised in Supplementary Table 1. Most of the studies are case-control studies nested within traditional prospective cohort studies, with some variation in the case mix of these studies according to the prevalence of prostate-specific antigen (PSA) testing within that population during follow-up. For example, there is a generally higher proportion of early stage and low grade cases in studies from the USA, where there has been relatively high levels of PSA-testing since the mid-1990s, than in studies in European populations where PSA-testing has only more recently started to become common. Two studies (PCPT and PLCO) are observational investigations using data from trials that included organised screening for prostate cancer. In these two studies, men with a raised PSA or abnormal digital rectal examination at recruitment-screening were excluded, and the eligible cases were diagnosed during subsequent follow-up. In PCPT the majority of cases were diagnosed at the end of the study and 7 years after recruitment and were detected through routine end of study biopsy. In PLCO the majority of cases were detected by routine PSA screening.

Collaborators were asked to provide data on concentrations of testosterone and SHBG, and on a number of other selected hormones and nutritional biomarkers. The majority of the studies were matched case-control studies nested within either prospective cohort studies[5-8, 11, 14-16, 18] or randomised trials[1, 4, 9, 10, 20-23]. Of the randomised trials, data were available for participants in both the intervention and placebo arm in ATBC, CARET, HHS, and PHS. Assay data were only collected in the screening arm of the PLCO trial[23], and the placebo arm in PCPT (as the intervention arm was designed to alter prostate hormone concentrations)[20]. Four studies were cohort or case-cohort analyses (BLSA, HIMS, MCCS, MMAS)[2, 3, 12, 13, 17, 19], in which hormone concentrations had been measured from stored serum from all, or a subset, of the cohort. To apply a consistent statistical approach across all studies, the cases from the case-cohort studies were matched to up to four participants who were free of prostate cancer at the age at diagnosis of the case on the basis of our minimal matching criteria (Supplementary Table 2). Some studies used density sample, meaning that an individual could appear more than once in a data file.

Individual participant data were also contributed for participant characteristics including: age, height, weight, smoking status, alcohol consumption, marital status, education achievement, ethnicity, and PSA and insulin like growth factor-I (IGF-I) concentrations at blood collection (where available). Collaborators also provided information on assay and time and date of blood collection. Information was requested about prostate cancer included date of diagnosis and stage and grade of disease. Men were excluded from the analyses if data were missing for date of birth, blood collection, or diagnosis (for cases) or if they were known to be receiving androgen therapy at blood collection.

**Data Processing**

Prostate cancer cases were defined as early stage if they were tumour–node–metastasis (TNM) stage ≤T2 with no reported lymph node involvement or metastases, or stage I-II, or equivalent (i.e., a tumour that does not extend beyond the prostate capsule); advanced stage if they were TNM stage T3 or T4 and/or N1+ and/or M1, stage III–IV, or equivalent (i.e., a tumour extending beyond the prostate capsule and/or lymph node involvement and/or distant metastases); or “unknown” otherwise. Aggressive disease was categorised as "no" for TNM stage ≤T3 with no reported lymph node involvement or metastases or equivalent, "yes" for TNM stage T4 and/or N1+ and/or M1 and/or stage IV disease or death from prostate cancer, or “unknown” otherwise. Prostate cancer was defined as low-intermediate grade if the Gleason score was <8 or equivalent (i.e., extent of differentiation good, moderate, or poor), high grade if the Gleason score was ≥8 or equivalent (i.e., undifferentiated), or grade “unknown” otherwise.

**Statistical analyses**

Free testosterone concentration was categorised into study-specific tenths, with cut-points defined by the distribution in control participants. Study-specific tenths were calculated to allow for any systematic differences between the studies in assay methods and blood sample types[24]. These differences mean that **absolute clinical cut-points cannot be used, but study-specific categorisation of concentrations will allow for relative concentrations to be determined within studies[25].**

**Estimates of prostate cancer risk were calculated using logistic regression conditioned on the matching variables and adjusted for**: age at blood collection (continuous), BMI (<25, 25.0-27.4, 27.5-29.9, ≥30.0 kg/m^2^, unknown (6%)), height (≤170.0, 170.0-174.9, 175.0-179.9, ≥180.0 cm, unknown (6%)), usual alcohol consumption (none, 1-9, 10-19, 20-39, ≥40 g ethanol/day, unknown (32%)), smoking status (never, former, current, unknown (10%)), marital status (yes, no, unknown (23%)), education status (<secondary/high school, secondary/high school, university, unknown (24%)). These categories were selected *a priori* based on previous evidence of associations with prostate cancer risk reported in the literature, with cut-points based on measurement availability and data distributions. **The highest tenth was used as the** reference category.

To explore the association with greater power, these tenths were also grouped (1, 2-4, 5-7, 8-10), with the 8^th^-10^th^ tenths combined as the reference category. Then, in all further analyses, the 2^nd^-10^th^ tenths were combined and used as the reference category. Where more than two categories of exposure were compared, variances were used to calculate floating confidence intervals, which facilitate comparisons between any two exposure groups[26, 27].

In the HPFS, assays were carried out in three distinct phases; therefore phase-specific tenths were calculated within this study (Supplementary Table 3).

PSA, IGF-I and C-peptide concentrations at blood collection were available for subsets of participants. The main analyses were repeated and the relationships between low free testosterone and prostate cancer risk were examined in these subsets before and after further adjustment for these variables (log transformed PSA (continuous) and study-specific fifths of IGF-I, and C-peptide concentrations).

***Tests for heterogeneity in the odds ratio estimates***

Heterogeneity in the associations of free testosterone with prostate cancer risk among studies was assessed by comparing the χ² values for models with and without a study*analyte interaction term. This was tested across between all studies as well as in studies which included organised screening in their study design (PCPT and PLCO).

Heterogeneity in the associations of free testosterone with prostate cancer risk was tested by: grade at diagnosis (low; high), stage at diagnosis (localised; advanced), aggressiveness of disease (not aggressive; aggressive), age at diagnosis (<65, 65+ years), years from blood collection to diagnosis (<5, 5+ years), age at blood collection (<65; 65+ years), date of diagnosis (pre 1990, 1990 onwards), PSA at blood collection (<2 ng/mL or ≥2 ng/mL), educational attainment status (no university degree; university degree), BMI (<30, 30+ kg/m^2^), smoking status (former/never; current), and alcohol consumption (<10, 10+ g ethanol/day), currently married/cohabiting (yes; no), IGF-1 concentration (<study specific median; study specific median+), time of blood collection (before 12:00; after 12:00), and testosterone assay (extracted; not extracted).

Tests for heterogeneity for case-defined factors, in which controls in each matched set were assigned to the category of their matched case, were obtained by fitting separate models for each subgroup and assuming independence of the ORs using a χ^2^ test, which is analogous to a meta-analysis. Tests for heterogeneity for non-case defined factors were assessed with χ² tests of interaction between subgroups and the binary variable.

***Statistical software***

All statistical tests were carried out with Stata statistical software, release 14.1 (StataCorp, College Station, TX, USA).

**Supplementary tables and figures**

**Supplementary Table 1: Sample populations, recruitment and case ascertainment methods**

|  | Sample population | Location | Recruitment period | Age at blood collection (years) | Prostate cancer ascertainment method |
| --- | --- | --- | --- | --- | --- |
| ATBC | Randomised trial of α-tocopherol and β-carotene among smokers | Finland | 1985-1993 | 50-69 | Cancer registry linkage, central review of medical records and specimens |
| BLSA | Prospective cohort study of the physiology of aging | USA | 1958-onward | 30-84 | Self-report with medical record review |
| CARET | Randomised controlled trial of β-carotene and retinyl palmitate in heavy smokers and asbestos workers | USA | 1987-1998 | 47-77 | Self-report with medical record review |
|  |  |  |  |  |  |
|  |  |  |  |  |  |
| CHDS | Prospective cohort study | USA | 1959-1966 | 20-55 | Cancer registry linkage |
| EPIC | Prospective cohort study | Europe | 1991-2001 | 43-76 | Cancer registry linkage; health insurance record linkage; self-report with medical record review |
| EPIC- Norfolk | Prospective cohort study | UK | 1991-1997 | 48-77 | Cancer registry linkage |
| FMC | Prospective cohort study | Finland | 1966-1972 | 17-80 | Cancer registry linkage |
| HHS NBSBWG | Randomised controlled trial of gemfibrozil | Finland | 1981-1982 | 40-56 | Cancer registry linkage |
| HIMS | Population-based cohort study | Australia | 1996-1999 (Blood collection 2001-2004) | 71-87 | Cancer registry linkage |
| HPFS | Cohort study of male dentists, optometrists, osteopathic physicians, podiatrists, pharmacists, and veterinarians | USA | 1986 | 46-87 | Self-report with medical record review |
| JACC | Prospective cohort study | Japan | 1988-1990 | 57-85 | Cancer registry linkage |
| JPHC | Prospective cohort study | Japan | Cohort 1)1990-1992  Cohort 2)1993-1995 | 41-71 | Active patient notification from major local hospitals in the study area and linkage with population-based cancer registries. |
| Janus NBSBWG | Prospective cohort study | Norway | 1973-onward | 33-61 | Cancer registry linkage |
| MCCS | Prospective cohort study | Australia | 1990-1994 | 40-72 | Cancer registry linkage |
| MEC | Prospective cohort study | USA | 1993-1996 (Blood collection 2001-2006) | 48-85 | Cancer registry linkage |
| MMAS | Prospective cohort study of the physiology of aging | USA | 1987-1989 | 41-70 | Cancer registry linkage |
| NSHDC | Combination of a population-based intervention study to decrease cardiovascular disease and a population-based monitoring study of cardiovascular disease | Sweden | 1985-onward | 39-61 | Cancer registry linkage |
|  |  |  |  |  |  |
|  |  |  |  |  |  |
| PCPT | Randomised, placebo-controlled trial of finasteride and prostate cancer | USA | 1994-1997 | 55-83 | Diagnosed as part of trial protocol. Annual digital rectal examinations and PSA measurements. Biopsy if abnormal DRE or reported PSA level > 4.0 ng per. End-of-study prostate biopsy |
| PHS | Randomised trial of aspirin and β-carotene among physicians | USA | 1982-onward | 41-78 | Self-report with medical record review |
|  |  |  |  |  |  |
|  |  |  |  |  |  |
| PLCO | Randomised controlled multicentre trial for early detection of cancer of the prostate, lung, colorectum and ovary | USA | 1993-2001 | 55-74 | Medical and pathology record review after screening and self-report with medical record review |

Abbreviations: ATBC=The Alpha-Tocopherol, Beta-Carotene Cancer Prevention Study; BLSA= The Baltimore Longitudinal Study of Aging; CARET =The Carotene and Retinol Efficacy Trial; CHDS= Child Health and Development Studies; DRE= digital rectal exam; EPIC= European Prospective Investigation into Cancer and Nutrition ; FMC= Finnish Mobile Clinic Health Examination Survey*;* HHS= Helsinki Heart Study; HIMS=Health In Men Study; HPFS*=* Health Professionals Follow-up Study; JACC= Japan Collaborative Cohort Study; JPHC= Japan Public Health Center-based Prospective Study; JHCS= Japan-Hawaii Cancer Study; MCCS=Melbourne Collaborative Cohort Study; MEC= Multiethnic Cohort Study of Diet and Cancer; MMAS=Massachusetts Male Aging Study; NBSBWG=Nordic Biological Specimen Biobank Working Group; NSHDC=Northern Sweden Health and Disease Cohort; PCPT= Prostate Cancer Prevention Trial; PHS=Physicians' Health Study; PLCO= Prostate, Lung, Colorectal and Ovarian Cancer Screening Trial; PSA= prostate-specific antigen

**Supplementary Table 2: Criteria used by individual studies to match case patients and control subjects**

| Study | Case:control ratio | Age at recruitment | Date of recruitment | Time of blood draw | Other matching criteria |
| --- | --- | --- | --- | --- | --- |
| ATBC | 1:2 | ±1 y | ±28 days |  | Trial intervention group. study centre |
| BLSA* | 1:1 | ±6 mo |  |  | Follow-up time |
| CARET | 1:1 | ±5 y | Month from enrolment to blood draw, year of randomisation | ±2 h | Study centre, ethnicity |
| CHDS | 1:2 | ±1 y |  |  | Ethnicity |
| EPIC | 1:1 except for Umea centre (1:2) | ±6 mo |  | ±1 h | Recruitment centre, time between blood draw and last food or drink consumption, follow-up time |
| EPIC- Norfolk | 1:2 | ±3 y | ±3 mo |  | Follow-up time |
| FMC | 1:2 | Nearest available | ±1 mo |  | Municipality |
| HHS NBSBWG | 1:4 | ±2 y | ±2 mo |  | Study centre |
| HIMS* | 1:4 | ±1 y |  |  | ±1 y date of blood collection  Fasting status, diabetes, controls must be 'alive and at risk' beyond the case's date of diagnosis |
| HPFS | 1:1 | Year of birth ±1 y | Exact year | Midnight–9 am; 9 am–12 pm; 12 pm–4 pm; and 4 pm–midnight | PSA test before blood draw, season. control subjects had at least one PSA test after the date of blood draw |
| JACC | 1:3 | As close as possible |  |  | Recruitment area |
| JPHC | 1:2 | ±3 y | ±60 days | ±3 h | Municipality, duration of fasting at blood collection (±3 h) |
| Janus NBSBWG | 1:4 | ±2 y | ±6 mo |  | County of residence and Red Cross blood donor status (Oslo) |
| MCCS* | 1:3 | ±5 y | ±2 y |  | Assay batch, country of birth |
| MEC | 1:2 | ±1 y | ±6 mo | ±2 h | Geographic site, ethnicity, fasting status (<6, 6-7, 8-9, 10+ hours) |
| MMAS* | 1:4 | ±2 y | ± 2 y |  | Ethnicity |
| NSHDC | 1:2 | ±6 mo | ±2 mo |  | County of residency |
| PCPT | 1:1 | As close as possible | As close as possible |  | PCPT treatment arm (placebo only). All non-whites controls were sampled and then backfilled with whites to achieve frequency matching on age and family history. Controls were required to have completed end of study biopsy procedure. |
| PHS | 1:2 | ±1 y |  |  | Had not had a total or partial prostatectomy and smoking status |
| PLCO | 1:1 | ±5 y | Exact year |  | Follow-up time |

Abbreviations: ATBC=The Alpha-Tocopherol, Beta-Carotene Cancer Prevention Study; BLSA= The Baltimore Longitudinal Study of Aging; CARET =The Carotene and Retinol Efficacy Trial; CHDS= Child Health and Development Studies; EPIC= European Prospective Investigation into Cancer and Nutrition ; FMC= Finnish Mobile Clinic Health Examination Survey*;* HHS= Helsinki Heart Study; HIMS= Health In Men Study; HPFS*=* Health Professionals Follow-Up Study; JACC= Japan Collaborative Cohort Study; JPHC= Japan Public Health Center-based Prospective Study; JHCS= Japan-Hawaii Cancer Study; MCCS=Melbourne Collaborative Cohort Study; MEC= Multiethnic Cohort Study of Diet and Cancer; MMAS=Massachusetts Male Aging Study; NBSBWG=Nordic Biological Specimen Biobank Working Group; NSHDC=Northern Sweden Health and Disease Cohort; PCPT=Prostate Cancer Prevention Trial; PHS=Physicians' Health Study; PLCO=Prostate, Lung, Colorectal and Ovarian Cancer Screening Trial; PSA= prostate-specific antigen.

*Used a case–cohort design that was subsequently converted into nested case–control design.**Supplementary Table 3: Assay methods and geometric mean testosterone and SHBG concentrations by study**

| Study |  |  | Testosterone (nmol/L) | | |  | SHBG (nmol/L) | | |
| --- | --- | --- | --- | --- | --- | --- | --- | --- | --- |
|  |  |  | Method | CV % | Geometric mean (95% CI) |  | Method | CV % | Geometric mean (95% CI) |
| ATBC | Case |  | E RIA | 5.5^†^ | 20.1 (18.7-21.6) |  | IRMA | 4.2^†^ | 82.6 (75.9-89.8) |
|  | Control |  |  |  | 20.5 (19.5-21.5) |  |  |  | 85.2 (80.4-90.4) |
| BLSA | Case |  | NE RIA | 3.3-6.4^§^ | 15.0 (13.9-16.1) |  | NE RIA | 1.8-22^§^ | 75.9 (69.6-82.6) |
|  | Control |  |  |  | 15.3 (14.2-16.4) |  |  |  | 81.1 (74.6-88.2) |
| CARET | Case |  | E RIA | 1-12^§^ | 13.5 (12.9-14.1) |  | IRMA | 2-7^§^ | 27.5 (26.1-28.9) |
|  | Control |  |  |  | 14.1 (13.5-14.7) |  |  |  | 27.1 (25.7-28.5) |
| CHDS | Case |  | E RIA | 9-11^‡^ | 22.0 (21.1-23.0) |  | IA | 4-6^‡^ | 32.7 (31.1-34.3) |
|  | Control |  |  |  | 22.1 (21.4-22.7) |  |  |  | 32.9 (31.8-34.1) |
| EPIC | Case |  | NE RIA | 10.8-14.8^†^ | 15.9 (15.4-16.5) |  | IRMA | 7.7-12.2^†^ | 41.3 (39.7-43.1) |
|  | Control |  |  |  | 15.6 (15.1-16.2) |  |  |  | 42.9 (41.2-44.7) |
| EPIC Norfolk | Case |  | ECIA | 7.1-12.0^§^ | 15.9 (14.6-17.4) |  | ECIA | 4.6-5.7^§^ | 40.7 (36.7-45.1) |
|  | Control |  |  |  | 16.5 (15.5-17.5) |  |  |  | 43.5 (40.5-46.7) |
| FMC | Case |  | NE RIA | 4.5-7.2^‡^ | 23.5 (22.1-24.9) |  | IMF | 6.6-8.7^‡^ | 51.4 (47.9-55.2) |
|  | Control |  |  |  | 23.0 (22.0-24.0) |  |  |  | 50.5 (48.0-53.1) |
| HHS NBSBWG | Case |  | NE RIA | 5.5-13^§^ | 20.0 (18.4-21.7) |  | IMF | 1.3-10.1^§^ | 50.5 (45.7-55.7) |
|  | Control |  |  |  | 19.7 (18.9-20.6) |  |  |  | 50.2 (47.7-52.9) |
| HIMS | Case |  | LC-MS/MS | <6^‡^ | 12.3 (11.8-12.8) |  | IA | <7^‡^ | 38.9 (37.0-40.9) |
|  | Control |  |  |  | 12.1 (11.8-12.3) |  |  |  | 39.6 (38.6-40.6) |
| HPFS | Case |  | ECIA | ≤5.2^†¶^ | 15.5 (15.0-15.9) |  | IRMA | ≤11.5^†¶^ | 56.9 (55.0-58.9) |
|  | Control |  |  |  | 15.4 (15.0-15.9) |  |  |  | 56.9 (55.0-58.9) |
| JACC | Case |  | NE RIA | 5-12^*^ | 16.1 (14.2-18.1) |  | IRMA | 5.6-6.9^*^ | 40.4 (35.0-46.6) |
|  | Control |  |  |  | 15.7 (14.5-16.9) |  |  |  | 43.7 (40.0-47.8) |
| Janus NBSBWG | Case |  | E RIA | 5.5-13.0^§^ | 15.4 (14.6-16.2) |  | IMF | 5-15^§^ | 46.0 (43.2-49.1) |
|  | Control |  |  |  | 15.7 (15.1-16.3) |  |  |  | 47.5 (45.5-49.7) |
| JPHC | Case |  | ECIA | 1-3^§^ | 21.7 (20.9-22.4) |  | IRMA | 2-8^§^ | 45.9 (44.0-47.8) |
|  | Control |  |  |  | 22.3 (21.9-22.7) |  |  |  | 47.7 (46.7-48.6) |
| MCCS | Case |  | ECIA | 1.6^*^ | 15.3 (14.8-15.8) |  | IA | 6 ^*^ | 37.3 (35.9-38.8) |
|  | Control |  |  |  | 15.4 (15.0-15.8) |  |  |  | 36.6 (35.6-37.6) |
| MEC | Case |  | E RIA | 3.5^†^ | 18.2 (17.6-18.9) |  | ECIA | 3^†^ | 35.3 (33.9-36.9) |
|  | Control |  |  |  | 18.3 (17.8-18.7) |  |  |  | 36.0 (34.9-37.0) |
| MMAS | Case |  | E RIA | 4.6-7.2^§^ | 16.6 (15.6-17.6) |  | Filtration assay | 8-10.9^§^ | 30.3 (28.2-32.5) |
|  | Control |  |  |  | 16.4 (15.9-16.9) |  |  |  | 30.4 (29.4-31.5) |
| NSHDC | Case |  | N/S |  | 20.2 (19.4-21.0) |  | IRMA | N/S | 42.0 (40.1-44.0) |
|  | Control |  |  |  | 19.5 (18.8-20.3) |  |  |  | 40.9 (39.1-42.8) |
| PCPT | Case |  | ECIA | 7.6-11.9^§^ | 12.6 (12.3-12.9) |  | ECIA | 5.2-12.2^§^ | 36.7 (35.7-37.7) |
|  | Control |  |  |  | 12.4 (12.1-12.7) |  |  |  | 36.6 (35.6-37.6) |
| PHS | Case |  | NE RIA | 8.7^†^ | 16.0 (15.2-16.9) |  | IRMA | 8.9^†^ | 19.9 (18.7-21.1) |
|  | Control |  |  |  | 16.0 (15.4-16.6) |  |  |  | 20.6 (19.7-21.5) |
| PLCO | Case |  | NE RIA | 14^*^ | 16.2 (15.7-16.6) |  | IRMA | 18^*^ | 42.9 (41.5-44.4) |
|  | Control |  |  |  | 15.9 (15.5-16.3) |  |  |  | 44.0 (42.7-45.3) |

Abbreviations: ATBC=The Alpha-Tocopherol, Beta-Carotene Cancer Prevention Study; BLSA= The Baltimore Longitudinal Study of Aging; CARET =The Carotene and Retinol Efficacy Trial; CV=coefficient of variation; E RIA=extraction radioimmunoassay; ECIA= electrochemiluminescence immunoassay; EPIC= European Prospective Investigation into Cancer and Nutrition; FMC= Finnish Mobile Clinic Health Examination Survey; HHS= Helsinki Heart Study; HIMS= Health In Men Study; HPFS= Health Professionals Follow-Up Study; IA=immunoassay; IMF=immunofluorometry; IRMA= immunoradiometric assay; JACC= Japan Collaborative Cohort Study; JPHC= Japan Public Health Center-based Prospective Study; LC-MS/MS= Liquid chromatography-tandem mass spectrometry; MCCS=Melbourne Collaborative Cohort Study; MEC= Multiethnic Cohort Study of Diet and Cancer; MMAS=Massachusetts Male Aging Study; NBSBWG=Nordic Biological Specimen Biobank Working Group; NE RIA=non-extraction radioimmunoassay; NSHDC=Northern Sweden Health and Disease Cohort; PCPT= Prostate Cancer Prevention Trial; PHS=Physicians' Health Study; PLCO= Prostate, Lung, Colorectal and Ovarian Cancer Screening Trial.

* Not specified

†Intra-assay

‡ Inter-assay

§Intra-and inter-assay range

¶ Three separate assay phases

**Supplementary Table 4: Geometric mean free testosterone concentration by study-specific tenths (1^st^ vs 2^nd^-10^th^) and 1^st^ decile cut-point**

|  | Geometric mean (95% CI)  (pmol/L) | |  | 1^st^ decile cut-point (pmol/L) |
| --- | --- | --- | --- | --- |
| Study | 1^st^ tenth | 2^nd^ -10^th^ tenth |  |  |
| ATBC | 133 (116-152) | 232 (224-239) |  | 161 |
| BLSA | 83 (69-101) | 177 (170-184) |  | 102 |
| CARET | 170 (154-188) | 331 (322-339) |  | 218 |
| CHDS | 246 (225-269) | 509 (499-519) |  | 300 |
| EPIC | 127 (116-139) | 297 (291-303) |  | 155 |
| EPIC- Norfolk | 159 (133-191) | 301 (290-314) |  | 199 |
| FMC | 176 (155-199) | 410 (399-422) |  | 241 |
| HHS NBSBWG | 171 (149-196) | 334 (324-345) |  | 214 |
| HIMS | 97 (90-104) | 233 (230-237) |  | 147 |
| HPFS | 96 (89-103) | 225 (222-229) |  | 95 |
| JACC | 163 (130-206) | 288 (273-303) |  | 188 |
| JPHC | 130 (116-146) | 269 (262-275) |  | 170 |
| Janus NBSBWG | 179 (169-189) | 406 (401-411) |  | 230 |
| MCCS | 149 (139-160) | 314 (309-318) |  | 202 |
| MEC | 154 (143-166) | 397 (390-403) |  | 236 |
| MMAS | 152 (138-166) | 385 (376-393) |  | 210 |
| NSHDC | 169 (152-188) | 390 (381-398) |  | 235 |
| PCPT | 131 (124-139) | 248 (245-251) |  | 160 |
| PHS | 222 (199-247) | 439 (428-450) |  | 265 |
| PLCO | 133 (124-142) | 294 (290-299) |  | 170 |

Abbreviations: ATBC=The Alpha-Tocopherol, Beta-Carotene Cancer Prevention Study; BLSA= The Baltimore Longitudinal Study of Aging; CARET =The Carotene and Retinol Efficacy Trial; CHDS= Child Health and Development Studies; EPIC= European Prospective Investigation into Cancer and Nutrition; FMC= Finnish Mobile Clinic Health Examination Survey*;* HHS= Helsinki Heart Study; HIMS= Health In Men Study; HPFS*=* Health Professionals Follow-Up Study; JACC= Japan Collaborative Cohort Study; JPHC= Japan Public Health Center-based Prospective Study; JHCS= Japan-Hawaii Cancer Study; MCCS=Melbourne Collaborative Cohort Study; MEC= Multiethnic Cohort Study of Diet and Cancer; MMAS=Massachusetts Male Aging Study; NBSBWG=Nordic Biological Specimen Biobank Working Group; NSHDC=Northern Sweden Health and Disease Cohort; PCPT= Prostate Cancer Prevention Trial; PHS=Physicians' Health Study; PLCO= Prostate, Lung, Colorectal and Ovarian Cancer Screening Trial.

**References for the supplementary material**

[1] Dorgan JF, Albanes D, Virtamo J, Heinonen OP, Chandler DW, Galmarini M, et al. Relationships of serum androgens and estrogens to prostate cancer risk: results from a prospective study in Finland. Cancer Epidemiol Biomarkers Prev. 1998;7:1069-74.

[2] Carter HB, Pearson JD, Metter EJ, Chan DW, Andres R, Fozard JL, et al. Longitudinal evaluation of serum androgen levels in men with and without prostate cancer. Prostate. 1995;27:25-31.

[3] Parsons JK, Carter HB, Platz EA, Wright EJ, Landis P, Metter EJ. Serum testosterone and the risk of prostate cancer: potential implications for testosterone therapy. Cancer Epidemiol Biomarkers Prev. 2005;14:2257-60.

[4] Chen C, Weiss NS, Stanczyk FZ, Lewis SK, DiTommaso D, Etzioni R, et al. Endogenous sex hormones and prostate cancer risk: a case-control study nested within the Carotene and Retinol Efficacy Trial. Cancer Epidemiol Biomarkers Prev. 2003;12:1410-6.

[5] Tsai CJ, Cohn BA, Cirillo PM, Feldman D, Stanczyk FZ, Whittemore AS. Sex steroid hormones in young manhood and the risk of subsequent prostate cancer: a longitudinal study in African-Americans and Caucasians (United States). Cancer Causes Control. 2006;17:1237-44.

[6] Travis RC, Key TJ, Allen NE, Appleby PN, Roddam AW, Rinaldi S, et al. Serum androgens and prostate cancer among 643 cases and 643 controls in the European Prospective Investigation into Cancer and Nutrition. Int J Cancer. 2007;121:1331-8.

[7] Low Y-L, Taylor JI, Grace PB, Dowsett M, Folkerd E, Doody D, et al. Polymorphisms in the CYP19 Gene May Affect the Positive Correlations between Serum and Urine Phytoestrogen Metabolites and Plasma Androgen Concentrations in Men. J Nutr. 2005;135:2680-6.

[8] Heikkila R, Aho K, Heliovaara M, Hakama M, Marniemi J, Reunanen A, et al. Serum testosterone and sex hormone-binding globulin concentrations and the risk of prostate carcinoma: a longitudinal study. Cancer. 1999;86:312-5.

[9] Stattin P, Lumme S, Tenkanen L, Alfthan H, Jellum E, Hallmans G, et al. High levels of circulating testosterone are not associated with increased prostate cancer risk: a pooled prospective study. Int J Cancer. 2004;108:418-24.

[10] Manttari M, Elo O, Frick MH, Haapa K, Heinonen OP, Heinsalmi P, et al. The Helsinki Heart Study: basic design and randomization procedure. Eur Heart J. 1987;8 Suppl I:1-29.

[11] Platz EA, Leitzmann MF, Rifai N, Kantoff PW, Chen YC, Stampfer MJ, et al. Sex steroid hormones and the androgen receptor gene CAG repeat and subsequent risk of prostate cancer in the prostate-specific antigen era. Cancer Epidemiol Biomarkers Prev. 2005;14:1262-9.

[12] Hyde Z, Flicker L, McCaul KA, Almeida OP, Hankey GJ, Chubb SAP, et al. Associations between testosterone levels and incident prostate, lung, and colorectal cancer. A population-based study. Cancer Epidemiol Biomarkers Prev. 2012;21:1319-29.

[13] Chan YX, Alfonso H, Chubb SA, Handelsman DJ, Fegan PG, Hankey GJ, et al. Higher dihydrotestosterone is associated with the incidence of lung cancer in older men. Horm Cancer. 2017;8:119-26.

[14] Ozasa K, Nakao M, Watanabe Y, Hayashi K, Miki T, Mikami K, et al. Serum phytoestrogens and prostate cancer risk in a nested case-control study among Japanese men. Cancer Sci. 2004;95:65-71.

[15] Sawada N, Iwasaki M, Inoue M, Sasazuki S, Yamaji T, Shimazu T, et al. Plasma testosterone and sex hormone-binding globulin concentrations and the risk of prostate cancer among Japanese men: a nested case-control study. Cancer Sci. 2010;101:2652-7.

[16] Vatten LJ, Ursin G, Ross RK, Stanczyk FZ, Lobo RA, Harvei S, et al. Androgens in serum and the risk of prostate cancer: a nested case-control study from the Janus serum bank in Norway. Cancer Epidemiol Biomarkers Prev. 1997;6:967-9.

[17] Severi G, Morris HA, MacInnis RJ, English DR, Tilley W, Hopper JL, et al. Circulating steroid hormones and the risk of prostate cancer. Cancer Epidemiol Biomarkers Prev. 2006;15:86-91.

[18] Gill JK, Wilkens LR, Pollak MN, Stanczyk FZ, Kolonel LN. Androgens, growth factors and risk of prostate cancer: the Multiethnic Cohort. Prostate. 2010;70:906-15.

[19] Mohr BA, Feldman HA, Kalish LA, Longcope C, McKinlay JB. Are serum hormones associated with the risk of prostate cancer? Prospective results from the Massachusetts Male Aging Study. Urology. 2001;57:930-5.

[20] Thompson IM, Goodman PJ, Tangen CM, Lucia MS, Miller GJ, Ford LG, et al. The influence of finasteride on the development of prostate cancer. N Engl J Med. 2003;349:215-24.

[21] Gann PH, Hennekens CH, Ma J, Longcope C, Stampfer MJ. Prospective study of sex hormone levels and risk of prostate cancer. J Natl Cancer Inst. 1996;88:1118-26.

[22] Andriole GL, Crawford ED, Grubb RL, 3rd, Buys SS, Chia D, Church TR, et al. Prostate cancer screening in the randomized Prostate, Lung, Colorectal, and Ovarian Cancer Screening Trial: mortality results after 13 years of follow-up. J Natl Cancer Inst. 2012;104:125-32.

[23] Hayes RB, Reding D, Kopp W, Subar AF, Bhat N, Rothman N, et al. Etiologic and early marker studies in the prostate, lung, colorectal and ovarian (PLCO) cancer screening trial. Control Clin Trials. 2000;21:349s-55s.

[24] Key TJ, Appleby PN, Allen NE, Reeves GK. Pooling biomarker data from different studies of disease risk, with a focus on endogenous hormones. Cancer Epidemiol Biomarkers Prev. 2010;19:960-5.

[25] Hsing AW, Stanczyk FZ, Bélanger A, Schroeder P, Chang L, Falk RT, et al. Reproducibility of serum sex steroid assays in men by RIA and mass spectrometry. Cancer Epidemiol Biomarkers Prev. 2007;16:1004-8.

[26] Easton DF, Peto J, Babiker AG. Floating absolute risk: an alternative to relative risk in survival and case-control analysis avoiding an arbitrary reference group. Stat Med. 1991;10:1025-35.

[27] Plummer M. Improved estimates of floating absolute risk. Stat Med. 2004;23:93-104.
